# Analysis of retinal cell development in chick embryo by immunohistochemistry and *in ovo *electroporation techniques

**DOI:** 10.1186/1471-213X-10-8

**Published:** 2010-01-20

**Authors:** Sung Tae Doh, Hailing Hao, Stephanie C Loh, Tapan Patel, Haim Y Tawil, David K Chen, Anna Pashkova, Andy Shen, Huimin Wang, Li Cai

**Affiliations:** 1 Department of Biomedical Engineering, Rutgers University, 599 Taylor Road, Piscataway, NJ 08854, USA; 2 Institute of Cognitive Neuroscience, East Normal University, Shanghai, PR China

## Abstract

**Background:**

Retinal cell development has been extensively investigated; however, the current knowledge of dynamic morphological and molecular changes is not yet complete.

**Results:**

This study was aimed at revealing the dynamic morphological and molecular changes in retinal cell development during the embryonic stages using a new method of targeted retinal injection, *in ovo *electroporation, and immunohistochemistry techniques. A plasmid DNA that expresses the green fluorescent protein (GFP) as a marker was delivered into the sub-retinal space to transfect the chick retinal stem/progenitor cells at embryonic day 3 (E3) or E4 with the aid of pulses of electric current. The transfected retinal tissues were analyzed at various stages during chick development from near the start of neurogenesis at E4 to near the end of neurogenesis at E18. The expression of GFP allowed for clear visualization of cell morphologies and retinal laminar locations for the indication of retinal cell identity. Immunohistochemistry using cell type-specific markers (e.g., Visinin, Xap-1, Lim1+2, Pkcα, NeuN, Pax6, Brn3a, Vimentin, etc.) allowed further confirmation of retinal cell types. The composition of retinal cell types was then determined over time by counting the number of GFP-expressing cells observed with morphological characteristics specific to the various retinal cell types.

**Conclusion:**

The new method of retinal injection and electroporation at E3 - E4 allows the visualization of all retinal cell types, including the late-born neurons, e.g., bipolar cells at a level of single cells, which has been difficult with a conventional method with injection and electroporation at E1.5. Based on data collected from analyses of cell morphology, laminar locations in the retina, immunohistochemistry, and cell counts of GFP-expressing cells, the time-line and dynamic morphological and molecular changes of retinal cell development were determined. These data provide more complete information on retinal cell development, and they can serve as a reference for the investigations in normal retinal development and diseases.

## Background

The vertebrate retina contains seven major cell types, six neuronal and one glial. These cells are derived from multipotent retinal stem/progenitor cells. Previous studies have revealed that the development of the vertebrate retina is a conserved process of cell genesis with the following order of cell birth: ganglion cells, horizontal cells, cone photoreceptors, amacrine cells, bipolar cells, rod photoreceptors, and Müller glia. Similar to other parts of the central nervous system, the retina contains a layered structure with photoreceptors (rods and cones) located in the outer nuclear layer (ONL), short projection neurons (bipolar cells) and local circuit neurons (horizontal and amacrine cells) in the inner nuclear layer (INL), and long projection neuron (ganglion cells) in the ganglion cell layer (GCL) [1]. During early stages of retinal development, the outer neuroblastic layer (ONBL) consists almost entirely of mitotic progenitor cells, while newborn neurons (mostly consisting of amacrine and ganglion cells) reside in the inner neuroblastic layer (INBL). The position of mitotic progenitors within the ONBL varies depending upon their progress through the cell cycle, with S phase cells found on the vitreal side of the ONBL near the border with the INBL and M-phase cells found on the scleral side of the ONBL abutting the retinal pigment epithelium [2,3].

An important aspect in understanding retinal anatomy and function is to trace the development of various cell types during embryonic stages. Although significant progress has been made, a complete developmental process underlying retinal cell differentiation during embryonic development is still lacking. Previous studies have provided information of retinal development on the rate of progression through the cell cycle [4-7], the mode of cell divisions, e.g., symmetrical versus asymmetrical [8-15], cell migration [16], and the order of cell birth [2,3,17-20]. A cell is born when it withdraws from the cell cycle and undergoes differentiation. These studies are mainly based on DNA synthesis analysis using tritiated-thymidine (^3^H-TdR) or 5'-bromon-2'deoxy-uridine (BrdU) labeling methods. ^3^H-TdR or BrdU is incorporated into the genomic DNA of stem/progenitor cells during the S-phase of cell cycle before they withdraw from the cell cycle and undergo differentiation. These methods are particularly useful in determining the start and end of cell genesis. In addition, using cell type-specific markers, the onset of differentiation can be determined by identifying the earliest time points for which immunolabeling is observed [21,22]. However, a major drawback to these methods is that DNA replication occurs in the nuclei thus only the nuclei of the labeled cells are observed. In addition, many cell type-specific markers also label only the nuclei of cells. Cell type-specific markers may be able to distinguish between cellular subtypes but fail to reveal the subtle morphological differences that determine key functional differences. Furthermore, morphological changes were observed in previous studies of retinal degenerative diseases caused by mutation or loss of gene function [23,24]. Thus, important morphological information of the whole cell that accompanies molecular changes is critical to understanding normal development and disease states.

Here, we report studies aimed at revealing dynamic morphological and molecular changes in retinal cell development of the chick embryo. A plasmid DNA that expresses green fluorescent protein (GFP) as a marker was directly delivered into the embryonic chick subretinal space and electric pulses were applied to facilitate DNA uptake by retinal stem/progenitor cells using a rapid and convenient *in ovo *electroporation technique. With this technique, GFP-expressing plasmids were efficiently transfected into retinal stem/progenitor cells with little damage to the chick embryos. GFP expression has been found in all cell types of the developing chick retina and allowed for clear visualization of cell morphologies. Immunohistochemistry was performed to further confirm retinal cell types with specific molecular markers. By tracking the cell counts of various cell types based on cellular morphology, laminar location, and molecular markers, the composition of various cell types of the developing retina at different stages has been determined. Thus, this study provides more complete insight into both the morphological and molecular changes during chick embryonic retinal development.

## Results

The chick embryo has been the most advanced model organism suitable for experimental embryology and for studying the development of higher vertebrates [25]. In this report, the chick retina was used for the study of cellular morphological and molecular changes during embryonic development using *in ovo *electroporation and immunohistochemistry techniques. All results reported in this study were focused on the central portion of the developing chick retina.

### Onset and expression pattern of retinal cell type-specific markers

**Figure 1:**
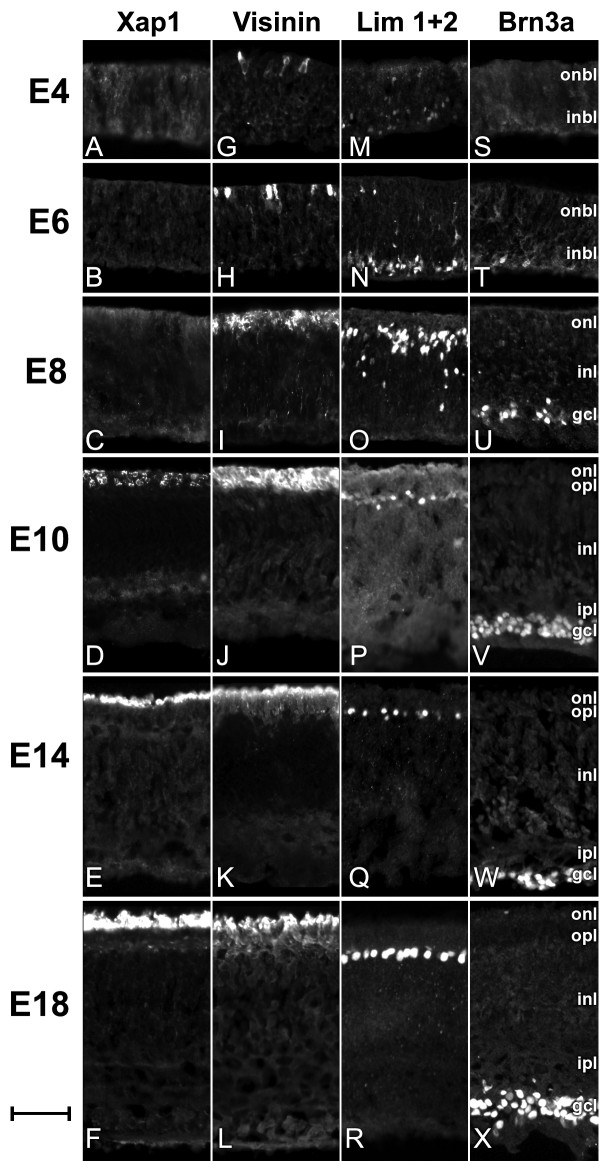
**The expression of chick retinal cell-type specific marker determined by immunohistochemistry method**. Retina tissue from chicken embryos were harvested at various time points during development, sectioned, and stained with retinal cell type specific antibodies Xap-1 (A-F), Visinin (G-L), Lim1+2 (M-R), and Brn3a (S-X). Xap-1 is known to selectively stain only the outer segments of photoreceptor cells, while Visinin is known to selectively stain the entire photoreceptor cells. Lim1+2 labels horizontal cells exclusively. Brn3a selectively labels as subset of ganglion cells. By staining retinas at various times during development, the onset of each cell type specific marker and their changes through out development were observed. ONBL, outer neuroblastic layer; INBL, inner neuroblastic layer; ONL, outer nuclear layer; INL, inner nuclear layer; GCL, ganglion cell layer. Scale bar = 40 μm.

To determine the onset of the marker expression of various cell types in the embryonic chick retina, cell type-specific antibodies, e.g., Visinin [26] and Xap-1 [27] for photoreceptors; Lim1+2 for horizontal cells [28-32], and Brn3a for ganglion cells [21,33-35] were used to stain retina sections harvested at various time points during retinal development from E4 to E18 (Fig. 1). The development of the many retinal cell types could be tracked independently by observing the onset and dynamic changes in expression patterns of these cell type-specific markers as detected by immunofluorescence labeling.

The expression of a photoreceptor marker Xap-1 [27,36-38] was observed only in the outer segment of the outer nuclear layer (ONL), and its expression starts sometime between E8 and E10 (Fig. 1A-D). The intensity of Xap-1 labeling continued to increase through E18 (Fig. 1E-F). The expression of another photoreceptor marker Visinin, a retinal photoreceptor protein which is believed to be cone specific [22,26,39], starts around E4 (Fig. 1G) which is much earlier than Xap-1 expression. At E6, its expression had increased in intensity but individual cells were still distinguishable (Fig. 1H). Visinin labeling then increased in intensity and the labeled cells composed a significant portion of the ONL at E8 (Fig. 1I). The intensity of Visinin labeling continued to increase and peaked at about E10 (Fig. 1J) when it was expressed in the entire ONL. In later stages (Fig. 1K-L), Visinin labeling continued to remain strong in the outer segment but diminished in the inner segment of the ONL. The differences in expression may suggest that Xap-1 and Visinin coincide with different stages of photoreceptor development. Other photoreceptor specific antibodies against Recoverin and Xap-2 did not show immunoreactivity in any of the stages (E4-E18) of chicken retina tested (results not shown).

The expression of a horizontal cell marker Lim1+2 [28-31] started in the retinal neural epithelium around E4 (Fig. 1M). A few Lim1+2 positive cells were observed in the inner neuroblastic layer (INBL) where newborn neurons appear. At E6 (Fig. 1N) the majority of Lim1+2 positive cells are near the edge of INBL, while some are migrating through the inner nuclear layer (INL) towards the outer portion of the INL where mature horizontal cells reside. Migration of Lim1+2 positive cells continues through E8 (Fig. 1O) and nears completion by E10 (Fig. 1P) when cells begin to align as a single layer adjacent to the ONL where synaptic endings of the photoreceptor cells located. By E14 (Fig. 1Q), the majority of Lim1+2 positive cells align on the outer portion of the INL. The space between the Lim1+2 positive horizontal cells were almost evenly spaced. This pattern was observed in all the later stages during chick retinal development. The horizontal cells at E18 (Fig. 1R) appear to be more mature than previous stages with larger and more oval cell bodies and are better aligned to the outer most region of the INL.

The expression of a retinal ganglion cell marker Brn3a [21,34,35] was observed to start in the GCL around E6 (Fig. 1T). Brn3a positive cells were organized into 3-4 cell layers in thickness by E8. Brn3a expression increased significantly in both intensity and in number of cells that expressed Brn3a at E8 (Fig. 1U), and its expression was restricted to the GCL. This increasing trend of staining continued at E10 (Fig. 1V) when the majority of cells in the GCL were Brn3a-positive. The borders of the INL, GCL, and the optic nerve fiber layer were clearly defined by Brn3a expression. From E10 (Fig. 1V) to E18 (Fig. 1X), Brn3a expression remained constant. The expression pattern of Brn3a at E21 (data not shown) was similar to that observed from E10 to E18.

**Figure 2:**
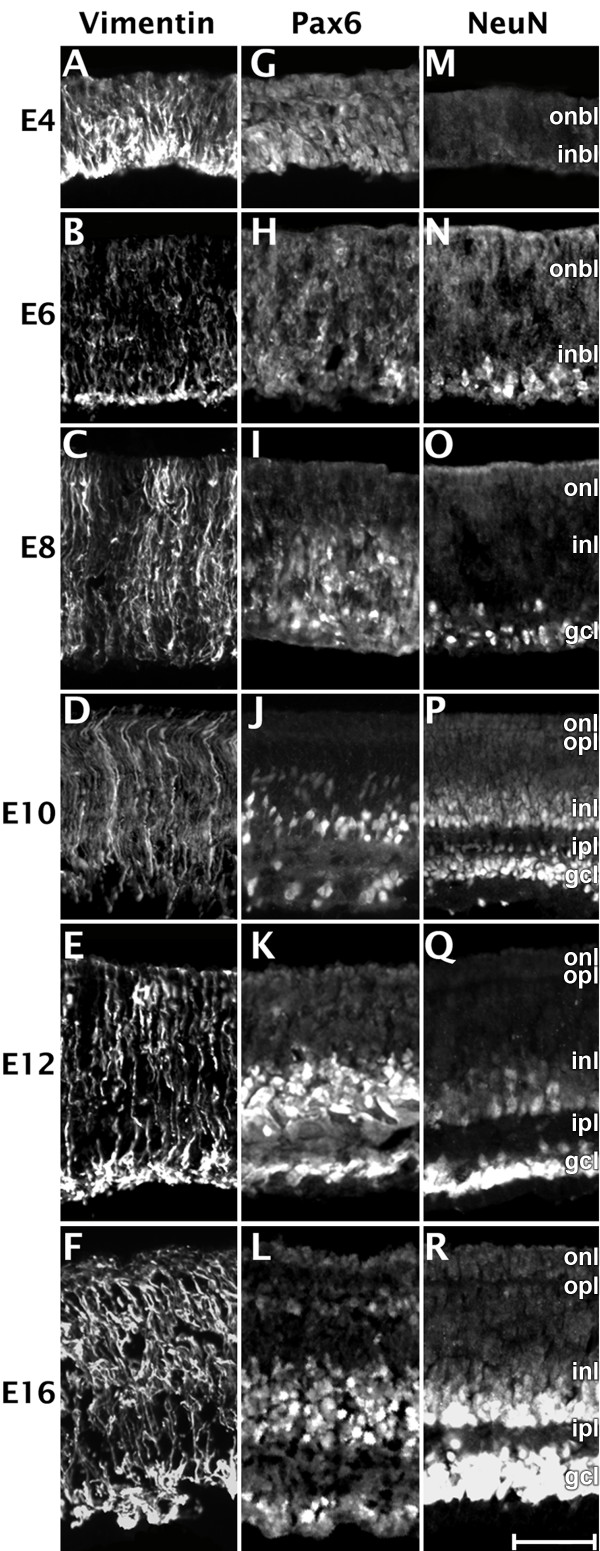
**The expression of glial and neuronal specific markers in the developing retina**. Retina tissue from chicken embryos were harvested at various time points during development, sectioned, and stained with antibodies Vimentin (A-F), Pax6 (G-L), and NeuN (M-R). While these markers are not cell-type specific, they label proteins that are involved in retina development. Vimentin labels radial glia in retina at early embryonic stages and Müller glia cells in retina at late embryonic stages. The Vimentin labeling resulted in the characteristic striated banding that stretched across the layers of the retina. Pax6 labels horizontal, amacrine, and ganglion cells. NeuN is a marker of early neurons and in the retina labels amacrine and ganglion cells. ONBL, outer neuroblastic layer; INBL, inner neuroblastic layer; ONL, outer nuclear layer; INL, inner nuclear layer; GCL, ganglion cell layer. Scale bar = 40 μm.

In addition, the expression pattern of neuronal specific markers, e.g., Pax6 and NeuN, and a radial glial cell/progenitor cell marker, Vimentin, were also examined (Fig. 2). Vimentin is an intermediate filament protein that is responsible for maintaining cell integrity [40] and is used to label radial glial cell/progenitor cell and Müller glia cells in the chicken retina [41,42]. Müller glia cells span all the retinal layers, possess radially polarized processes, and have arborizations called "end-feet" toward the GCL [41]. Vimentin positive cells were found in all stages tested (Fig. 2A-F) and were not seen to be specific to any cell layer. The Vimentin positive cells showed distinct striated banding throughout all layers. Immunoreactivity was usually most intense in the GCL, which is likely caused by the high concentration of processes in the end-feet of radial/Müller glial cells. Pax6 is a nuclear marker for ganglion, amacrine, and progenitor cells that is required for multipotency in retinal cells [42]. Pax6 was first detected at E4 in a small number of progenitor cells located in the INBL (Fig. 2G). Over the next few days, the overall number of cells detected increased and by E8 (Fig. 2I) included cells located in the INL. At E10 (Fig. 2J), ganglion cells, amacrine cells, and migrating cells are all clearly labeled by Pax6. By E16 (Fig. 2l), as found in previous reports, some horizontal cells are also labeled but labeling is weaker than that found in amacrine or ganglion cells [43]. NeuN is a neuron-specific nuclear protein marker [44]. The onset of immunoreactivity of this marker indicates terminal differentiation of the neuron. Previously, use of NeuN antibodies in the mouse retina showed immunoreactivity in the GCL and to a much lesser extent in the INL [44]. In the chicken retina, NeuN labeling is seen in the INBL beginning at E6 (Fig. 2N), and in the INL at E8 (Fig. 2O). By E10 (Fig. 2P), the INL and GCL can be very easily distinguished by the NeuN expression pattern. The intensity of NeuN expression was more intense and widespread in the GCL than the INL at E12 (Fig. 2Q). By E16 (Fig. 2R), the entire GCL and amacrine cell portion of the INL were labeled by NeuN.

### Morphological analysis of developing chick retina using in ovo electroporation technique

**Figure 3:**
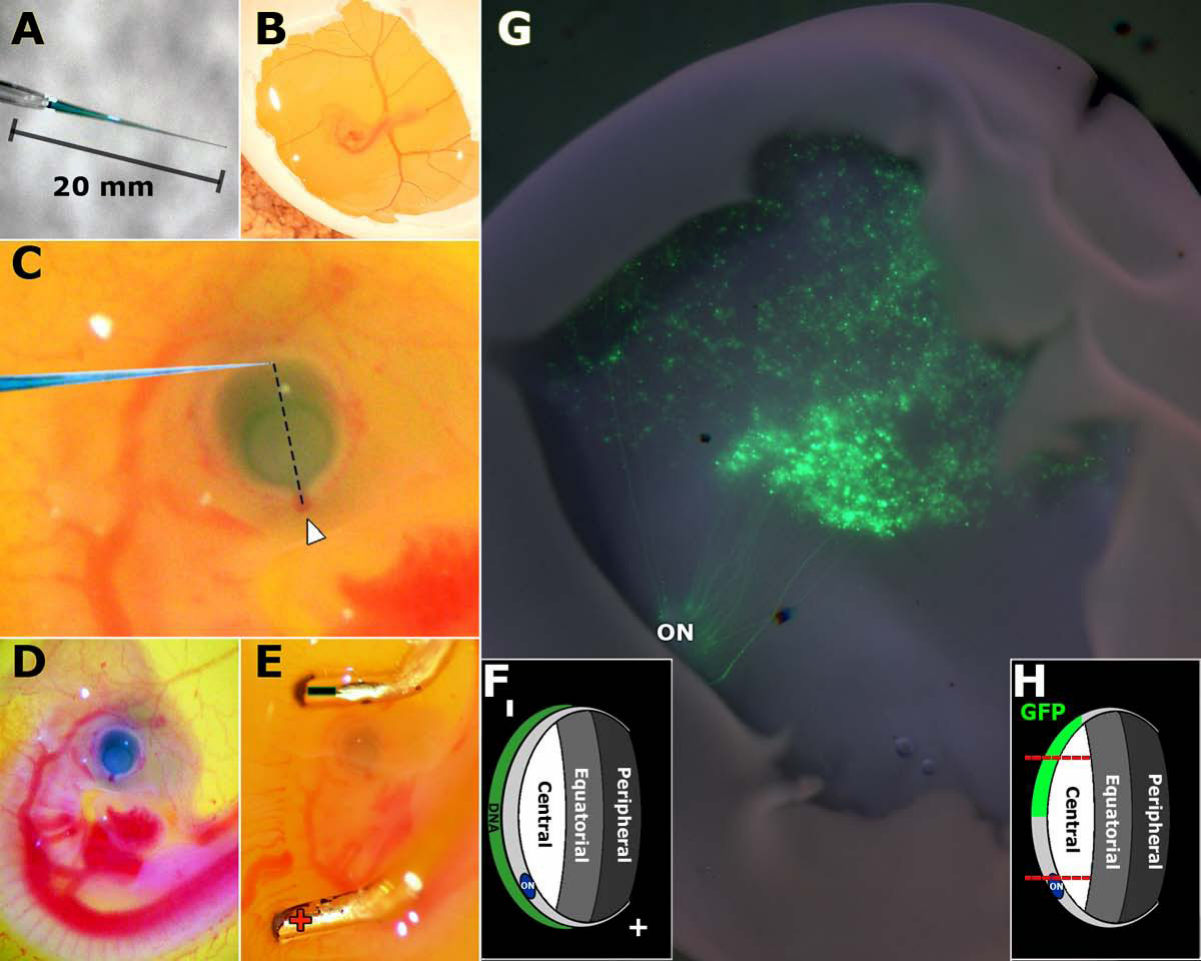
***In ovo *electroporation method targeting E3-E4 chicken retina**. Glass capillary tubes were pulled to fabricate needles with a tip opening at 0.1 μm in diameter and a 20 mm taper (A). The needle is loaded with DNA/0.025% fast green solution. Eggs were rotated to release the embryo and the shells were sterilized by wiping with 70% ethanol then windowed using forceps (B). The trajectory of the needle approached the eye from behind the head, toward the beak, and tangent to the retina surface (C). The outermost region of the retina opposite of the main bundle of blood vessels entering the eye (arrowhead, C) was targeted for injection. Successful injection was verified by observing that the subretinal space of the eye was filled with DNA/fast green solution (D). Electroporation was performed with the negative electrode placed above the head of the embryo and deeper in the albumin than the eye. The positive electrode was placed below the spine and on the surface of the albumin (E). Electroporation using this orientation drives the DNA in the subretinal space toward the positive electrode and into the retinal progenitor cells (F). The egg was sealed and incubated until tissue harvest at desired time points. Electroporated retinal tissues were then checked for GFP expression. A wholemount image of a retina with GFP expression at E14 (G) shows axons of ganglion neurons originating in the central retina and extending to the optic nerve (ON). Approximately 1/3 of the central retina was transfected with decreasing levels of GFP expression in more peripheral regions (G, H). Using this method of electroporation at E3-4, the central region of the developing chick retina (area between the two red dotted lines in H) was consistently and stably transfected with pCAG-GFP throughout *in ovo *developmental stages.

**Figure 4:**
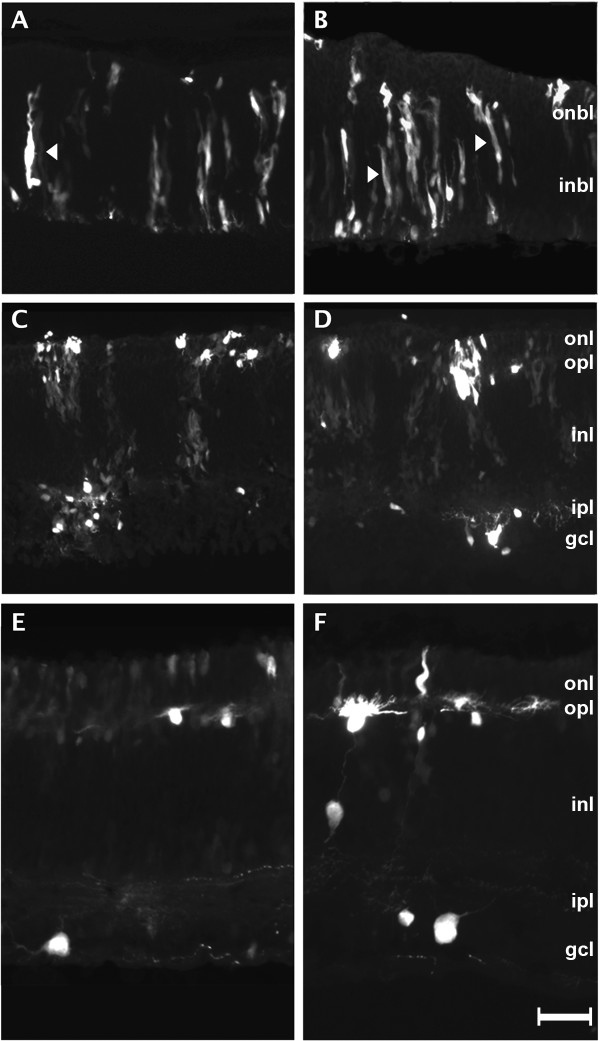
**Tracking development and migration of chicken embryonic retina cells using GFP labeling by *in ovo *electroporation technique**. Chicken embryos are injected with pCAG-GFP and electroporated at embryonic day 4 (E4). GFP expression is observed during early stages of development, E7-E8 (A-B). These cells are elongated which is characteristics of cell migration. The cells span the whole width of the neural epithelial layer. In subsequent stages E9-E10 (C-D), cell layers begin to show distinct boundaries and cells begin to settle into their final layers. Cells also take on a rounder morphology and begin to extend their processes. The appearance of well defined cell type specific morphologies begins around E12 (E). Processes are more clearly visible and help to form clearly visible boundaries between layers. The clearest and most distinct and definitive cell morphologies are observed in GFP-expressing cells at E18 (F) (see Fig. 4). ONBL, outer neuroblastic layer; INBL, inner neuroblastic layer; ONL, outer nuclear layer; INL, inner nuclear layer; GCL, ganglion cell layer. Scale bar = 50 μm.

**Figure 5:**
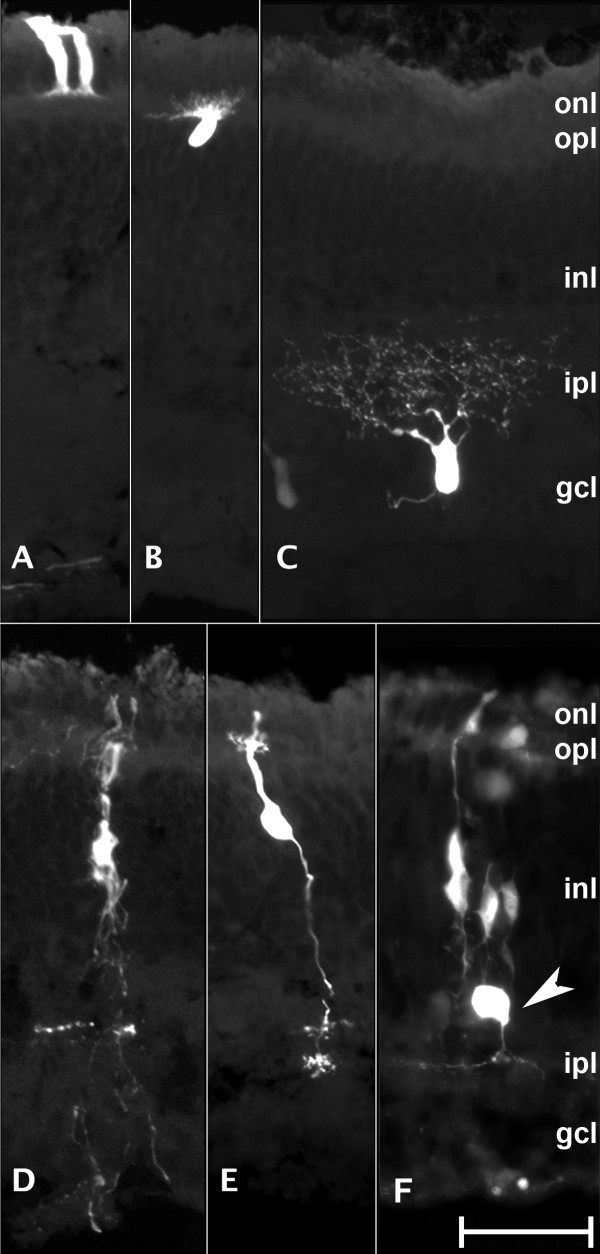
**Characteristic morphology of various cell types in chicken retina at E18 with GFP labeling by *in ovo *electroporation technique**. Expression of GFP is observed in all six cell types found in retina tissue through E18. Visualizing GFP expression at this stage shows cells localized in distinct layers and each of the cell type specific morphologies. Photoreceptors (A) have a cylindrical shape and are located in the outer nuclear layer (ONL). Horizontal cells (B) have processes located at the boundary of the inner nuclear layer (INL) and ONL with their cell bodies in the INL. Ganglion cells (C) are located in the GCL and have processes which mostly point toward the INL. Müller glial cells (D) span the entire retina with their cell bodies in the INL. Bipolar cells (E) have two distinct processes one that extends from the cell body in the INL to the ganglion cell layer (GCL) and the other to the ONL. Amacrine cells (F) have cell bodies in the INL and have processes that extent toward the GCL. OPL, outer plexiform layer; IPL, inner plexiform layer; Scale bar = 20 μm.

To reveal the dynamic morphological changes during retinal cell development, the *in vivo *electroporation method was adapted and optimized for chick retinal study (*in ovo *electroporation) [45,46]. Although *in ovo *electroporation is a widely used technique for the study of neural development, the technique has been mainly performed in neural tube injection and electroporation. Targeted retinal injection and *in ovo *electroporation at E3-4 is considered a novel method for the study of chick retinal development (See Methods section for technical detail). The pCAG-GFP DNA was injected into the sub-retinal space of E3-E4 chicken embryo (Fig. 3A-D) followed by electroporation (Fig. 3E-F). The plasmid DNA construct, pCAG-GFP, was previously shown to produce ubiquitous GFP expression without altering normal development [45]. *In ovo *electroporation of pCAG-GFP consistently resulted in the highest level of GFP expression in the central retina with decreasing GFP expression in more peripheral regions of the retina. Few if any cells in the peripheral retina were observed to express GFP (Fig. 3G-H). Immunohistochemistry revealed GFP expression in retinal stem/progenitor cells during early chick embryonic retinal development (Fig. 4A-B), and all six differentiated major cell types during late chick embryonic retinal development (Fig. 4C-F and Fig. 5). Visualization of cytoplasmic GFP expression revealed the cross section morphology of cell bodies and processes (axons and dendrites). The location of individual cells with respect to the retina layers was also clearly visible (Figs. 4 &5). The determination of a specific retinal cell type was based on the cellular morphology, laminar location, and expression of molecular markers of the cell.

By observing development between E7 (Fig. 4A) and E18 (Fig. 4F), the changes in laminar location were determined. During early development, the vast majority of cells are still proliferating and some have started their migration process (Fig. 4A-B). Migratory cells have elongated cell bodies that can span both the ONBL and INBL (arrows in Fig. 4A-B). Once migratory cells reach their respective laminar locations (Fig. 4C-E), they terminally differentiate into specific mature cell types. Differentiated cells (Fig. 4F and Fig. 5) have cell type-specific morphologies and more defined axons and dendrites. At E18, the characteristic morphologies and locations of all six major cell types found in a more developed retina were clearly seen through GFP labeling (Fig. 5). Visualization of GFP allows for the cell bodies as well as the processes (axons and dendrites) to be observed in great detail. Photoreceptor cells (Fig. 5A) are localized exclusively in the ONL and have elongated cell bodies like rods and cones that span the ONL. Their synaptic bodies are found along the boundary of the ONL and the outer plexiform layer (OPL). Horizontal cells (Fig. 5B) have oval cell bodies found in the region of the INL closest to the OPL. Their cell bodies align in a single cell layer with dendrites in the OPL that reach towards the ONL. Ganglion cells (Fig. 5C) are located in the GCL, their cell bodies seem to be the largest in all cell types in the retina. Their dendrites reach towards the INL and the inner plexiform layer (IPL). The size of their cell bodies and the number of dendrites can greatly vary depending on their subtype. Morphologically distinct cells (cell body size, process number, and process direction) were observed in the GCL at E18 (Fig. 4F, Fig. 5C). Based on the laminar location of these observed cells and the known morphological diversity among ganglion cells, it is concluded that multiple subtypes of ganglion cells were able to be labeled by this technique. Müller glial cells (Fig. 5D) are the principal glial cells of the retina. They form architectural support structures stretching radially across the thickness of the retina and are the limits of the retina at the outer and inner limiting membrane, respectively. They are also the least frequently found cell type in the retina accounting only about 2.7% of the total cell population in the mature mouse retina [2,3]. Their cell bodies sit in the INL and project thick and thin processes irregularly in either direction to the outer limiting membrane and the inner limiting membrane. Bipolar cells (Fig. 5E) have their cell bodies in the INL and reach from the ONL to the ganglion cell layer (GCL) but have only a single axon and dendrite in opposite directions. Amacrine cells (Fig. 5F) have round cell bodies found in the INL and have a single axon that extends to the inner plexiform layer (IPL) where it then branches out to contact the cells in GCL [47].

### Confirming retinal cell type of GFP-expressing cells by immunohistochemistry

**Figure 6:**
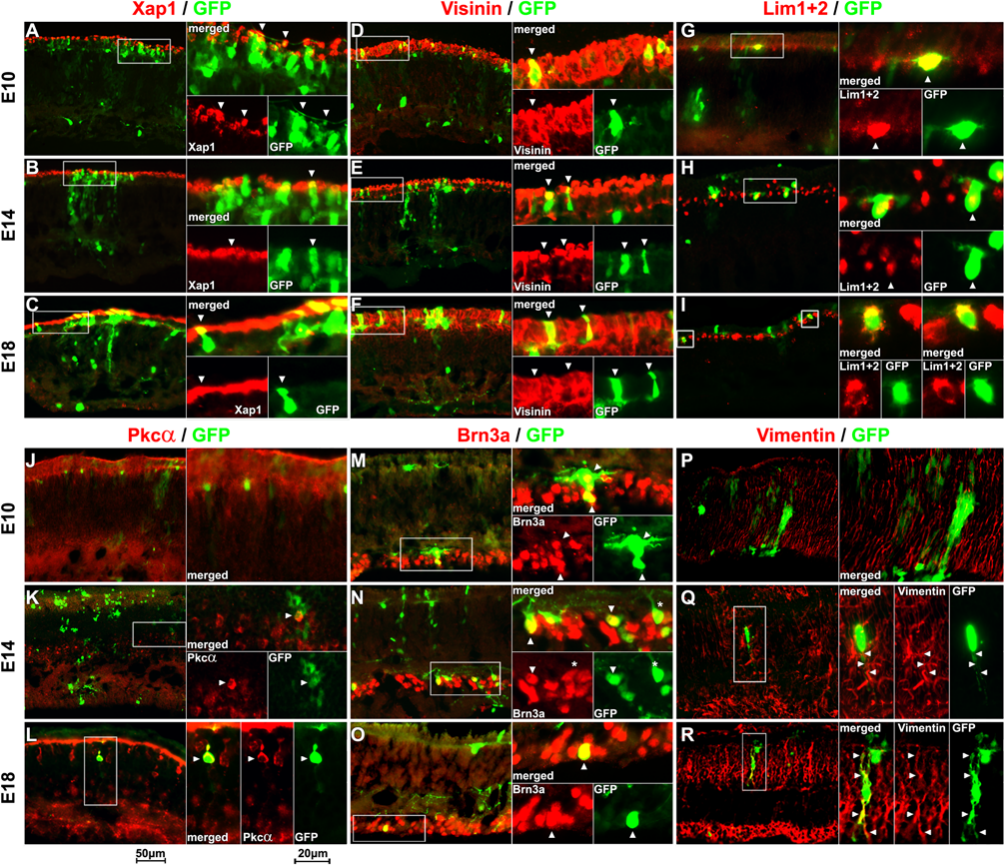
**Determine retinal cell type of the GFP-expressing cells using immunohistochemistry method**. GFP-expressing retinal tissues at three developmental stages (E10, E14, and E18) were sectioned and stained with retinal cell type specific antibodies, e.g., Xap-1 (A-C) and Visinin (D-F) for photoreceptor cells, Lim1+2 for horizontal cells (G-I), Pkcα for bipolar cells (J-L), Brn3a for ganglion cells (M-O), and Vimentin for Müller glial cells (P-R). For each set of images (A-R) the entire retina cross section is shown to allow for the laminar location to be easily visualized. The image on the right shows a merged high magnification image and a pair of separate images showing the antibody staining and GFP fluorescence. The white-boxed region is shown in higher magnification on the right. Double labeled cells are indicated by arrowheads at higher power. Staining with Xap-1 (A-C) and Visinin (D-F) confirmed the identity of GFP-expressing cells in the photoreceptor layer as cone photoreceptors. Lim1+2 labeling consistently labeled cells on the outermost region of the INL. Antibody staining with Pkcα failed to label any GFP-expressing cells at E10 (J). The Pkcα positive GFP-expressing cells were first seen at E14. Pkcα staining increased in both frequency and intensity in GFP-expressing cells at E18. Double labeled cells showed round cell bodies at both E14 and E18 and two distinct processes extending in opposite directions, characteristic of bipolar cells, were regularly observed at E18 (L, GFP). Antibody staining with Brn3a confirms that the GFP-expressing cells (arrowheads in M-O) are ganglion cells. As previously reported Brn3a does not label all ganglion cells. This was seen in E14 tissue (N) where GFP-expressing cells that show ganglion cell morphology and location but no Brn3a labeling (asterisk). Vimentin showed strong labeling at E10 (P) however failed to show double labeling with GFP. Double labeling at E14 (Q) was very rare and slightly more frequent at E18 (R). In both cases double labeling was only seen in the processes of the cells (Q-R, arrowheads).

To confirm that cell types were correctly identified by cellular morphology and laminar location and to determine composition of cell types of GFP-expressing cells, immunohistochemistry was performed on E10, E14 and E18 retina sections with pCAG-GFP transfection. The cell types were determined using the GFP images (green cells in Fig. 6) and confirmed by overlaying the images with cell type specific antibody labeling (red cells in Fig. 6). At E10, a large number of GFP-expressing cells have immature cell morphology (Fig. 6A, D, G, J, M, P). However, each cell type has a defined laminar location with cell migration close to completion. The laminar location combined with the morphological characteristics (described above and shown in Fig. 5) allowed for the cell types to be quite accurately determined. The identification of each retinal cell type at E14 (Fig. 6B, E, H, K, N, Q) became even easier as the vast majority of cells have already completed migration, and their characteristic morphologies, such as axons and dendrites, were more clearly defined. GFP-expressing cells were stained with a cell type specific antibody, e.g., Xap-1 (Fig. 6A-C) or Visinin (Fig. 6D-F) for photoreceptors, Lim1+2 (Fig. 6G-I) for horizontal cells, Pkcα (Fig. 6J-L) for bipolar cells, Brn3a (Fig. 6M-O) for ganglion cells, and Vimentin (Fig. 6P-R) for Müller glial cells. For photoreceptor cells, immunolabeling showed that Visinin and Xap-1 staining was only found in ONL where photoreceptor cells reside. The results of antibody labeling with GFP-expressing cells showed that cells in ONL were positive with Visinin and Xap-1 staining. Visinin and Xap-1 labeled all photoreceptors in the ONL beginning at E10. Xap-1 did not label the whole photoreceptor cell but as previously reported only the outer segment [27]. Xap-1 has been shown to be expressed by photoreceptors exclusively under conditions in which the outer segment membranes are properly assembled [36]. The fact that Xap-1 expression was observed in the outer most region of the outer nuclear layer (ONL) beginning at E8 and E10 may indicate that this outer most region stained by Xap-1 is the developing outer segment of the ONL. Our results further indicate that development of the outer segment of the ONL may start as early as E8. Each of the cells labeled with photoreceptor specific markers were correctly identified based on laminar location and cellular morphology (Fig. 6A-F). Lim1+2 staining was only found in the outer region of the INL. GFP-expressing cells in the outer border of the INL, with round cell bodies, showed Lim1+2 staining at each of the developmental stages. Pkcα has been shown to specifically label bipolar cells in the developing retina [48-50]. Pkcα labeling showed no staining at E10 (J). Staining at E14 (K) showed labeled cells intermittently in the INL. Staining became more frequent and more intense at E18 (L) in the INL where bipolar cells reside. Double labeling was first observed at E14 and became more frequent at E18. Double labeled cells were only observed in the INL, showed round cell bodies, and characteristic processes were regularly observed at E18. Brn3a staining was exclusively localized in GCL where ganglion cells reside. A few GFP-expressing cells that showed ganglion cell-type specific characteristics were not labeled with Brn3a (Fig. 6N, marked with an asterisk). However, this finding does not exclude this cell from being a ganglion cell as Brn3a was shown to label the majority of but not all ganglion cells [47]. Labeling with Vimentin showed striated banding throughout all layers for all the various stages. Double labeling with GFP and Vimentin was not observed at E10 but migrating cells expressing GFP seemed to follow Vimentin labeled cells. Double labeling was first seen at E14 (Q) and only in the processes of the cell (Q-R). At all three stages, individual whole cell bodies were resolved by GFP labeling, as opposed to only the outer segment (Fig. 6A-C), entire layers (Fig. 6D-F), or only the nuclei (Fig. 5 and 6M-O) as resolved using antibody labeling. The molecular identification of retinal cell types confirmed that the cell types of chick retina cells between E10 and E18 could be accurately determined based on cellular morphology and laminar location as revealed by GFP expression.

### Dynamic changes in the composition of GFP-expressing cell types during retinal development

**Figure 7:**
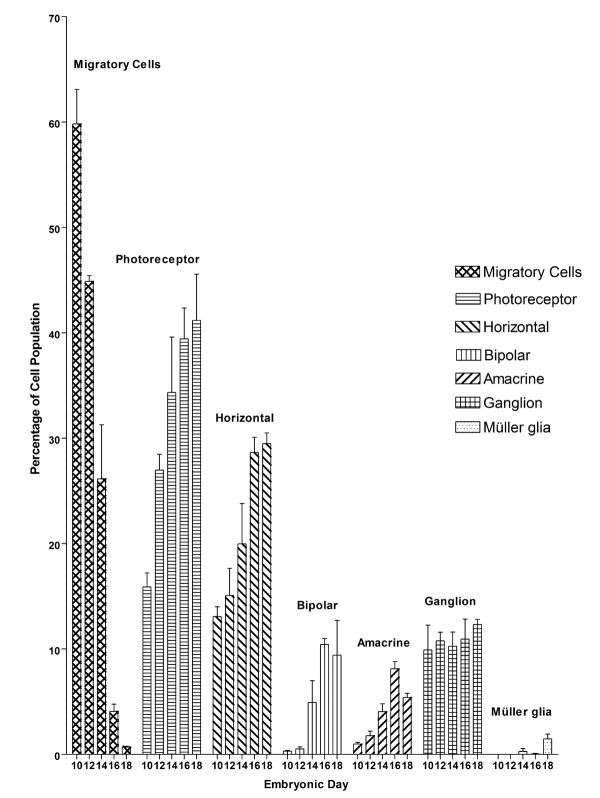
**Cellular composition at various stages during embryonic development of the chicken retina**. Three retina samples were collected every other day from E10 to E18. Retinal cells expressing GFP were categorized into one of seven cell types based on retinal laminar location, cellular morphology, and molecular marker. Cell counts of each cell type were used to determine the distribution of each cell type during development of the retina. The data shows that the number of ganglion cells remained fairly consistent throughout this time period. Photoreceptors and horizontal cells have a significant increase in population while the increase is less dramatic in bipolar and amacrine cells. As expected, with the increase in other cell types the number of migratory cells decreases.

To identify dynamic changes in the developing chicken retina, GFP-expressing cells were counted for each cell type from E10 to E18. At least three retinas with GFP expression were generated for each time point. The cell type of the GFP-expressing cells was determined, categorized, and counted. The percentage of each cell type among the entire population of GFP-expressing cells (composition of each retinal cell type) was calculated at each time point (Fig. 7). Cell types were determined based on their morphology, laminar location, and molecular marker. The cell counts showed a dramatic decrease in the number of migratory cells from E10 to E18 (Fig. 7). This finding indicates that the number of migrating cells decreased steadily as fewer migratory cells were being generated and more migratory cells differentiated during this time frame. By E18, the percentage of migratory cells was less than 1%, suggesting that cell migration almost reached completion. The largest increases in differentiated cell types occurred at E12 for photoreceptors, E14 for horizontal cells, and E16 for bipolar cells and amacrine cells. Ganglion cells did not show significant changes during these time periods. Morphologically mature Müller glia cells were first seen around E14 but still remained in very small proportion at E18.

By E18, the composition of retinal cell types is approximately 41.2% photoreceptor, 29.5% horizontal, 9.4% bipolar, 5.4% amacrine, 12.3% ganglion, 1.5% Müller glia, and 0.7% migratory cells (Fig. 7).

## Discussion and Conclusion

The timing of neurogenesis in the chicken retina was previously determined using [^3^H] tymidine autoradiographs by Prada et al. However, due to technical limitations, retinal cell development with dynamic morphological changes in relation to the changes in molecular markers was not fully determined. To address these limitations, we have adopted existing *in ovo *electroporation capabilities to develop a new method of *in ovo *electroporation that can specifically target retinal progenitor cells (E3-E4) resulting in the ability to visualize all six major retina cell types at the single cell level. Conventional methods (electroporation at E1.5) have difficulty labeling late-born neurons in the retina such as bipolar cells. This new method adds important capacities to allow possible future studies where the precise cell morphology of retinal neurons is required. It can also be applied to gain/loss of function studies where a gene of interest can be targeted to study normal development and/or disease of the retina. In this study, we have tracked the morphological and molecular development of each of the cell types in the developing chick retina and determined the relative abundance of each cell type within the total population over a developmentally critical time frame, thereby providing new insights into retinal development.

### Photoreceptors

Almost all vertebrate retinas have two morphological types of photoreceptors (rods and cones) that mediate dim-light, color vision and fine-detail detection [51]. Although autoradiographic studies fail to distinguish rods and cones in the chick retina [52,53], ultrastructural studies using scanning electron microscopy confirmed that both rods and cones do exist [54]. However, photoreceptor percentages vary with species, cones being a majority in the chick retina, i.e., 86% cones versus 14% rods [55]. In this study, the earliest time we observed photoreceptor marker Visinin expression was at E4 (Fig. 6G-L), which is about two days earlier than it has previously been reported at E6 [22]. Since Visinin preferentially labels cone photoreceptors [26,39], this suggests that cone photoreceptor development in chick begins early at about E4. For rods, antibodies against Rhodopsin and Recoverin were used to immunostain the developing chick retina from E6-E18. No labeling was detected with Rhodopsin and Recoverin antibodies (data not shown), suggesting that either the rod-specific antibodies were not specific to chick rods due to differences in species, or that chick rods differentiate after the examined time-frame (E6-E18).

### Horizontal and Amacrine cells

For the study of retinal horizontal cell and amacrine cell development, we used the antibody against Lim1+2 [28-30,56], Pax6 [43,57] and NeuN [58,59]. Transcription factor Lim1+2 is essential for horizontal cell development and its laminar position in the retina [28,30,60-63]. Pax6 homeobox gene is among the earliest genes expressed in the eye primordia and plays crucial roles in retina development [32,42,60]. It is also known to be an amacrine cell marker in later stages (after E8) during embryonic retinal development [43]. By E10, the cells labeled with Pax6 show three distinct layers consisting of migratory cells, amacrine cells, and ganglion cells which can be distinguished using their laminar location. Staining with Lim1+2 antibody showed that horizontal cells began differentiation as early as E4. Lim1+2 staining showed that the vast majority of Lim1+2 expressing horizontal cells completed migration to the outer region of the INL by E10. The dynamic expression pattern of Lim1+2 during horizontal cell differentiation in our study (Fig. 1M-R) is consistent with previously observations as the differentiating horizontal cells undergo bi-directional interkinetic nuclear migration [30,61]. Pax6 also weakly labeled some horizontal cells after E14. The staining patterns of Lim1+2 and Pax6 indicate that amacrine and horizontal cells can be distinguished from each other using laminar location information beginning at E10. The DNA-binding, neuron-specific protein NeuN, is present in most neuronal cell types of vertebrates. NeuN stained ganglion cells but labeled very few cells in the INL in adult human retinas [59] and in E12.5 mice [44]. We found that NeuN was strongly expressed in the majority of cells in both the GCL and INL, which is quite different from the one reported in mouse and human. It has been reported that NeuN staining in the adult chick retina is weaker in the INL than in the GCL [64]. This observation suggests that in amacrine cells, NeuN may be most highly expressed shortly after or during differentiation.

### Bipolar Cells

In cell counts of GFP-expressing cells, bipolar cells were not observed with significant frequency until E14. Immunolabeling with Pkcα antibody was also not observed until E14 supporting the cell count data. Birth-dating studies of bipolar cells in rodents showed bipolar cells are among the later born cell types being born postnatally along with Müller cells [2,3]. Consistent with previous studies [53], the bipolar cells in chick retina have been shown to be the last cell type to become postmitotic.

### Ganglion Cells

Previous studies show that ganglion cells are produced over the period from E2 to E9 [53], with all cells initially born in the ventricular zone, followed by immediate differentiation and migration into the future ganglion cell layer [65]. The development of ganglion cells begins at the central region of the developing retina, gradually spreading to the peripheral region as a wave-like front [66]. The composition of GFP-expressing ganglion cells in this report did not show significant changes from E10 to E18, indicating that they were generated before E10. Brn3a is a transcription factor that regulates the development, morphology, and function of retinal ganglion cells [21,34,35]. It is expressed specifically in the nuclei of cells that have finished migration and begun differentiating into ganglion cells. Previous findings have shown that Brn3a is expressed as early at E4.5 in the chick retina [33]. Combining the early onset of Brn3a antibody labeling and the consistent percentage of ganglion cells from E10 to E18, it is believed that the vast majority of ganglion cells complete development before E10. This observation is consistent with previous finding that all ganglion cells are born before E9 [53]. Since multiple subtypes of ganglion cells express GFP after *in ovo *electroporation of pCAG-GFP, the fact that most of the GFP-expressing ganglion cells were also Brn3a-positive (Figs. 1 &6A-F) indicates that Brn3a labels the majority of ganglion cell subtypes but not all ganglion cells [47].

### Müller Glial Cells

The birth dating of Müller glial cells has been controversial. Electron microscopic studies indicate they are born early [67,68]. However, results from ^3^H-TdR or BrdU labeling indicate that Müller glia cells are labeled only after injections were preformed at late stages during retina development. The immunolabeling results (Fig. 2A-F) show that there is constant expression of Vimentin throughout the embryonic period from E4 to E18. Vimentin is known to label radial glial cells, a type of progenitor cell in the central nervous system [69-71]. Müller glial cells are the only glial cell type in the retina suggesting that Vimentin-positive cells are either mature Müller glial cells in mature retina or retinal stem/progenitor cells in early embryonic retinal development. Radial glial cells or progenitors exist throughout the embryonic stages of retinal development and serve as structurally stabilizing scaffolds [72] and as cell migration guides [73]. GFP-expressing Müller glial cells with mature morphology (Fig. 5D, Fig. 6P-R) could only be observed after E14. The proportion of GFP-expressing Müller glial cells was small, e.g., under 0.3%, at E14 and gradually increased to only 1.5% at E18 (Fig. 6R and Fig. 7). The low number of morphologically mature Müller glia cells expressing GFP observed between E10 and E18 could be because 1) they are naturally scarce during this time frame or 2) the late birth of Müller glial cells makes them less compatible for labeling using the *in ovo *electroporation method described. First, the early onset and high frequency of immunoreactivity of Vimentin suggests that a significant number of radial glial/progenitor cells are born as early as E4 and maintain a significant population throughout the examined time frame. Second, if their late birth results in the lack of Müller glial cell labeling, bipolar cells should also be rarely seen, since bipolar cells are born around the same time as Müller glial cells. Third, GFP labeling at E18 showed similar proportions of retina cell population for ganglion cells (which are born early) and bipolar cell (which are born late). Therefore, we believe that the time of birth should not significantly affect the proportion of cell types observed at E18 unless those cells are not yet born. For these reasons, we conclude that the lack of cells with mature Müller glia morphology is not due to their inherent rarity or incompatibility with *in ovo *electroporation but that the majority of Müller glial cells do not exhibit morphological maturity before E18. A possible explanation for the lack of morphologically mature Müller glia are that they may maintain their progenitor cell (radial glia) state for a significant period of time (at least 14 days) after being born. If this is the case, then the cells that serve as scaffolds and migration guides for the migratory neurons in the retina are radial glia cells/progenitor cells in early embryonic development. In the mouse retina, the majority of Müller glia cells reach morphological maturity late in development [2,3]. It is known that many of Müller glial cells continue to differentiate postnatally in the chick retina [74]. Alternatively, the staining of Vimentin in microglia cells and proliferating precursor cells is another consideration [75]. Microglia cells are known to be present in small numbers during the development of retina and involved in clearing dying cells that are part of the normal developmental processes [76,77]. However, labeling of Vimentin in microglia cells may only slightly contribute to the early onset and high frequency of Vimentin labeling. Furthermore, neither microglia nor proliferating precursor cells can account for the radially polarized staining pattern.

In conclusion, this study reveals the dynamic morphological and molecular changes during a critical period of chick embryonic retinal development. We have demonstrated that *in ovo *electroporation with pCAG-GFP combined with immunohistochemistry is a very efficient technique for tracing cell proliferation, migration and differentiation processes during retinal development. We were able to identify specific retinal cell types of the GFP-expressing cells based on their morphology and laminar location. The cellular identity of GFP-expressing cells was further confirmed by immunostaining using cell type-specific antibodies. Although, this method has been used in study of retinal development, sustained reporter gene expression in the developing chicken retina has not reported to last for more than a few days [78,79]. *In ovo *electroporation at HH10 (~E2) targeting the optic vesicle is able to transfect cells that develop to form the eye. However, these cells have a very high turnover at this time and this method is not specific for retina cells. It may be that the high cell turnover rate prevents sustained stable expression. By E3, the embryo is developed enough that the major structures of the eye are all formed but young enough that the majority of cells in the retina are still retinal stem cells. The vitelline membrane is thin enough to allow for microinjection and the blood vessels are spaced far enough from the eye to allow for electroporation of the embryo without damaging the vessels. As demonstrated in this study, we were able to optimize the *in ovo *electroporation method to successfully transfect the retinal stem/progenitor cells at E3-E4 resulting in all 6 major retinal cell types to express GFP through E18. Furthermore, GFP expression clearly shows the cellular morphology that other techniques, e.g., ^3^H-TdR or BrdU labeling methods, failed to provide. In rodents, retinas injected and electroporated with pCAG-GFP at postnatal day 0 (P0) did not show GFP expression in early born cells, e.g., horizontal or ganglion cells, which indicates that the generation of these two types are completed by P0 in rodents. By visualizing the morphology of whole individual cells in the developing retina, characterization of each cell type can be performed dynamically during normal development, disease states, or specific over-expression of critical retinal genes. For example, this method can be applied to study the development and growth of the axons and dendrites of particular cell types or applied to produce sustained over-expression or knockdown of developmental genes in the chick retina using alternative DNA constructs. As shown in our results this method can be easily combined with well established immunohistochemistry methods which will be helpful for the understanding of the molecular events that accompany morphological changes during normal development or disease of the retina.

## Methods

### Chicken Embryos

Fertilized pathogen-free (SPF) white leghorn chicken (*Gallus domesticus*) eggs were obtained from Sunrise Farms (Catskill, NY). These eggs were incubated at 37.5°C and 60% humidity (GQF manufacturing, Savannah, GA) for 88-92 hours (~3 - 4 days) to obtain embryos that are at the developmental stage HH21. Stages of the chick embryo were determined according to Hamburger and Hamilton [80]. All of the animal experiments were approved by the Institutional Animal Care and Facilities Committee at Rutgers University.

### In Ovo Electroporation

Microinjections were performed using a micropipette needle made from pulled glass capillary tubes with a tip opening at about 0.1 μm in diameter and a 20 mm taper (Fig. 3A). Needles with larger tips have difficulty piercing the vitelline membrane while smaller tips have difficulty loading and delivering the DNA solution. The taper minimizes damage to the embryo while maintaining enough structural integrity in the needle for handling microinjections. The needles were attached to a 0.1 ml Hamilton Gastight 1710 syringe (Reno, NV) mounted on a WPI M3301-M3 micromanipulator (Sarasota, FL). The needle was loaded with a mixture of DNA (pCAG-GFP; 1.5 μl with concentration ranging 3.0-6.0 μg/μl) and 0.025% fast green dye (0.2 μl) to allow visualization of the injection (Fig. 3A). This amount of DNA loaded per needle is enough for about a dozen egg injections. The condition and location of the embryo can be seen by candling the egg. The vitelline membrane of the egg was freed from the inner membrane with gentle rotation. The egg was placed with the larger end up and windowed (Fig. 3B) as previously described [46] with minor changes being that the windowing was placed immediately above the air cell and albumen was not removed. The vitelline membrane was not removed as the needle was sharp enough to easily pierce through this membrane. Injection into the vitreous humor allows DNA to diffuse away from the retina and therefore requires more DNA to be injected or results in poor transfection. To maximize the travel of the needle point in the subretinal space, the needle should approach the eye such that it is almost at a tangent to the section of the retina targeted for transfection (Fig. 3C). To prevent critical damage to the brain or heart the needle was inserted by approaching from caudal to rostral direction towards the beak. The targeted injection site was along the dorsal region of the eye contralateral to the main bundle of blood vessels entering the eye (Arrowhead in Fig. 3C). When injecting at this angle, continually injecting the DNA while slowly retracting the needle allows visualization of the DNA/fast green solution either filling the subretinal space or the vitreous humor. Injection of the subretinal space can be verified by the filling of DNA/fast green solution following the outline of the eye (Fig. 3D) rather than diffusing away or filling into the middle of the eye. Every attempt was made to consistently target the same injection site for each embryo to minimize variation from retina to retina. The injection site was electroporated using a BTX ECM 830 electroporation system (Harvard Apparatus, MA). The BTX Genetrodes (model 514) were spaced 3-5 mm apart. The electrodes were placed in parallel so that the developing eye was situated between the electrodes (Fig. 3E). Electroporation with the electrodes placed in this manner transported DNA located in the subretinal space towards the positive electrode and into the retina (Fig. 3F) resulting in approximately half of the central retina being transfected (Fig. 3G-H). The electroporation settings were 5 pulses of 15 mV for 50 ms with 950 ms pauses between each pulse. After electroporation, the window on the operated eggs was sealed with clear scotch tape, and the egg was returned to the incubator.

### Tissue Processing and Sectioning

Chick embryos were harvested at various times after injection, electroporation, and placed in cold 1× PBS (Phosphate buffered saline, Fischer Scientific). Retinas were dissected at embryonic day 8 (E8) or older stages, while retinas younger than E8 were left intact in the embryo to minimize damage. Tissues were fixed by immersion in 4% paraformaldahyde (in 1× PBS) for 90 minutes at 4°C and then infiltrated overnight in 30% sucrose (in 1× PBS).

For retinas at E8 and older, the peripheral regions of the cryoprotected retinas were removed to ensure only the central region of the retina was included for analysis. The face of a clock will be used to describe the regions of the whole retina. The retina was oriented such that from an overhead view the dorsal region was oriented at 12 o'clock and the ventral region to the 6 o'clock position. Once situated in this orientation, a first cut is made from the 2 o'clock to the 10 o'clock positions. A second cut is made from the 4 o'clock to the 8 o'clock positions (red dotted lines in Fig. 3H). A third cut from the 1 o'clock to the 5 o'clock positions; and a final cut from the 7 o'clock to the 11 o'clock positions. The resulting square piece in the center is designated as central retina region (see Fig. S1A in additional file 1). In most of the cases, this central square region contained the vast majority of GFP expressing cells.

For retinas younger than E8, the whole eye was sectioned along with the head at the horizontal plane (see Fig. S1B in additional file 1). Only sections of the retina that contain the lens were used to ensure that the central region (CR) of the retina is analyzed. The central region of the retina was defined as the area that opposite to the lens as shown in the fig. S1B in additional file 1.

For embryos injected with pCAG-GFP, successful transfection (Fig. 3G) was verified by examining the retinas under a fluorescent dissection microscope, Leica MZ16FA (Leica Microsystems, Germany) before embedding and sectioning. Tissues were embedded in OCT (Electron Microscopy Sciences, Hatfield, PA) and stored at -80°C until ready for sectioning. Retina tissues sections at 10-15 μm were cut using a cryostat (Thermo 0620E), mounted on Superfrost slides (Fisher Scientific) and air-dried. Immunohistochemistry was performed immediately afterwards.

### Immunohistochemistry

For immunofluorescence staining, tissue sections were fixed in 4% paraformaldahyde for 5 minutes and washed in PBS. Blocking solution (175 μl; 0.05% Triton X-100, 10% goat serum, 3% BSA in PBS) was applied on the slide and incubated for 30 minutes at room temperature followed by washing in PBS. Primary antibodies XAP-1 [27] (100 μl of 1:10 dilution; DSHB, IA), Xap-2 [27] (100 μl of 1:100 dilution; DSHB, IA), Visinin [26] (100 μl of 1:10 dilution; DSHB, IA), rho-4D2 [81] (100 μl of 1:100 dilution; R.S. Molday, University of British Columbia), Lim1+2 (4F2) [56,82,83] (100 μl of 1:10 dilution; DSHB, IA), Vimentin (H5) [71] (100 μl of 1:10 dilution; DSHB, IA), Pax6 [57] (100 μl of 1:100 dilution; DSHB, IA), Pkcα (100 μl of 1:400 dilution; Santa Cruz Biotechnology Inc, CA), Recoverin [84] (100 μl of 1:100 dilution; Millipore, MA), Brn3a [21] (100 μl of 1:100 dilution; Millipore, MA), or NeuN [44] (100 μl of 1:1000 dilution; Millipore, MA) were applied to the wet slides. Incubation was carried out in a humidified box on a slow rocker at 4°C overnight. As a negative control, serum and secondary antibodies were applied but no primary antibody was added to the staining solution (Fig. S2 in additional file 1). Slides were then washed with PBST (0.1% Tween-20 in 1× PBS) and Cy3-conjugated secondary antibodies (150 μl of 1:300 dilution; Jackson ImmunoResearch, West Grove, PA) was applied. After 30 min incubation at room temperature with gentle rocking, the slides were washed with PBST then cover slipped. All washes were 5 min and repeated 3 times unless specified otherwise.

### Imaging

Microscopy and imaging analysis were performed using an upright fluorescence microscope (Zeiss Axio Imager A1) with a monochrome digital camera Axiocam MRM (Zeiss, Germany). Images of GFP-expressing cells and secondary antibody Cy3 labeled cells were taken separately using FITC and DsRed filters, respectively. Imaging of Vimentin-labeled retinas was performed using a confocal microscope (Nikon Eclipse 80i) with a monochrome digital camera Nikon D-Eclipse C1 (Nikon, Japan). Images of GFP-expressing cells and secondary antibody Cy3 labeled cells were taken separately using 488 nm and 543 nm wavelengths, respectively. Images of Cy3 and GFP channels were then overlaid using Adobe Photoshop CS to create pseudo-colored double-labeled images.

### Cell counts

Retinas electroporated with pCAG-GFP were harvested as described above at each time point (E10, E12, E14, E16, and E18). At least three retinas from each time point with confirmed GFP expression were then sectioned and imaged. To avoid counting cells that span multiple sections more than once, only one image was counted from any given set of 5 serial sections.

### Determining cell type for cell counts

In the majority of cases, the morphology and laminar location criteria are sufficient for the determination of a cell type. For photoreceptor cells, Visinin labeling starts in the ONBL as early as E4 and is restricted in the ONL from E8 and beyond. Furthermore no other cell types other than Müller cells were observed through antibody staining to be in the ONL. However, the cell body of Müller cells is generally not located in the ONL. Therefore, all cells restricted to only the ONL were identified as photoreceptors. Horizontal cells, in the time frame of E10-E18, were shown to be strictly restricted to the outermost region of the INL by Lim1+2 staining. The morphologies as revealed by GFP show round cell bodies with the majority of processes extending toward the OPL. Therefore cells with round cell bodies found in the outermost region of the INL that have processes generally restricted to the OPL were identified as horizontal cells. Ganglion cells were labeled with Brn3a and found only in the GCL between E10 and E18. The cell bodies of ganglion cells are also known to be round and among the largest of all the cell types in the retina. Therefore, cells located in the GCL with round cell bodies were identified as ganglion cells. There may be a small number of displaced amacrine cells that were counted as ganglion cells; however, we believe that this should not significantly affect the accuracy of our cell counts. Bipolar cells were known to be restricted to the INL (which was confirmed by staining with Pkcα) with round cell bodies and two distinct bi-directional processes. The greatest difficulty in identifying this cell type was in distinguishing bipolar cells from Müller cells and migratory cells as each of these cell types could have their cell bodies in the INL. We have found, however, that even in their progenitor state after E10 they do have subtle but distinct morphological characteristics that allow for them to be distinguished from each other. Müller glial cells have multiple branching processes extending from the cell body. Migratory cells have elongated cell bodies and are usually clustered with other migratory cells. Bipolar cells have rounder cell bodies and at most 2 processes which, if present, are transversely opposed from each other. Pax6 is known to label horizontal, amacrine, and ganglion cells. NeuN is known to label amacrine and ganglion cells. Comparing the staining patterns of these two markers between E10 and E18 shows that the inner half of the INL and the GCL are consistently labeled by both markers. Based on these staining patterns amacrine cells were determined to be restricted to the inner half of the INL between E10 and E18. Amacrine cells also have round cell bodies with processes that are directed toward the IPL. Therefore, all cells with round cell bodies, processes directed toward the IPL, and located in the inner half of the INL were counted as amacrine cells. In all cases characteristic laminar location of cell types narrows down the potential identity of the cell, while morphological characteristics which became increasingly distinguished over time increased the accuracy of cell type identification.

### Statistical Analysis

For each cell type, its percentage of the total GFP-expressing cells was calculated each retina. The data was then plotted and the standard error of the mean (SEM) was calculated for each set of retinas of the same time point using Prism version 4.03 (GraphPad Software, Inc. La Jolla, CA).

## Authors' contributions

SD participated in the design of the study, carried out the experiments, and drafted the manuscript. HH, SCL, TP, HYT, DC, AP, and AS helped immunohistochemistry and microscopy. AS, DKC, AP, and HW helped the cell counting. LC designed, coordinated, and carried out the experiments, and helped to write and revise the manuscript. All authors read and approved the final manuscript.

## Supplementary Material

Additional file 1 ***Fig. S1*. Diagram depicting the central region of the retina included for analysis**. A. For retinas at E8 and older, the peripheral regions of the cryoprotected retinas were removed to ensure only the central region of the retina was included for analysis. The face of a clock will be used to describe the regions of the whole retina. The retina was oriented such that from an overhead view the dorsal region was oriented at 12 o'clock and the ventral region to the 6 o'clock position. Once situated in this orientation, a first cut is made from the 2 o'clock to the 10 o'clock positions. A second cut is made from the 4 o'clock to the 8 o'clock positions (red dotted lines in Fig. 3H). A third cut from the 1 o'clock to the 5 o'clock positions; and a final cut from the 7 o'clock to the 11 o'clock positions. The resulting square piece in the center is designated as central retina region. B. For retinas younger than E8, the whole eye was sectioned along with the head at the horizontal plane. The central region (CR) of the retina was defined as the area that opposite to the lens. ***Fig. S2*. Negative control for antibody staining**. Retina tissue from chicken embryos were harvested at E4, E6, and E8, sectioned, and stained with only serum and secondary antibody. ONBL, outer neuroblastic layer; INBL, inner neuroblastic layer; ONL, outer nuclear layer; INL, inner nuclear layer; GCL, ganglion cell layer. Scale bar = 40 μm. Click here for file
