# Supplementary material for: Analysis of retinal cell development in chick embryo by immunohistochemistry and in ovo electroporation techniques
Source: BMC Dev Biol. 2010 Jan 20;10:8. doi: 10.1186/1471-213X-10-8 (PMC2822752; doi:10.1186/1471-213X-10-8)

Additional File

***Supplemental Figure 1*. Diagram depicting the central region of the retina included for analysis**

A. For retinas at E8 and older, the peripheral regions of the cryoprotected retinas were removed to ensure only the central region of the retina was included for analysis. The face of a clock will be used to describe the regions of the whole retina. The retina was oriented such that from an overhead view the dorsal region was oriented at 12 o'clock and the ventral region to the 6 o'clock position. Once situated in this orientation, a first cut is made from the 2 o'clock to the 10 o'clock positions. A second cut is made from the 4 o'clock to the 8 o'clock positions (red dotted lines in Fig. 3H). A third cut from the 1 o'clock to the 5 o'clock positions; and a final cut from the 7 o'clock to the 11 o'clock positions. The resulting square piece in the center is designated as central retina region.

B. For retinas younger than E8, the whole eye was sectioned along with the head at the horizontal plane. The central region (CR) of the retina was defined as the area that opposite to the lens.

***Supplemental Figure 2*. Negative control for antibody staining**

Retina tissue from chicken embryos were harvested at E4, E6, and E8, sectioned, and stained with only serum and secondary antibody. ONBL, outer neuroblastic layer; INBL, inner neuroblastic layer; ONL, outer nuclear layer; INL, inner nuclear layer; GCL, ganglion cell layer. Scale bar = 40 μm.

Supplemental Fig. 1

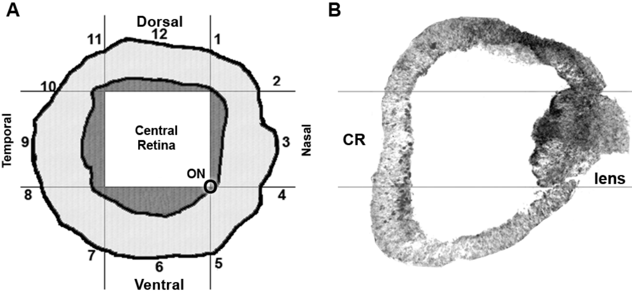

Supplemental
Fig. 2

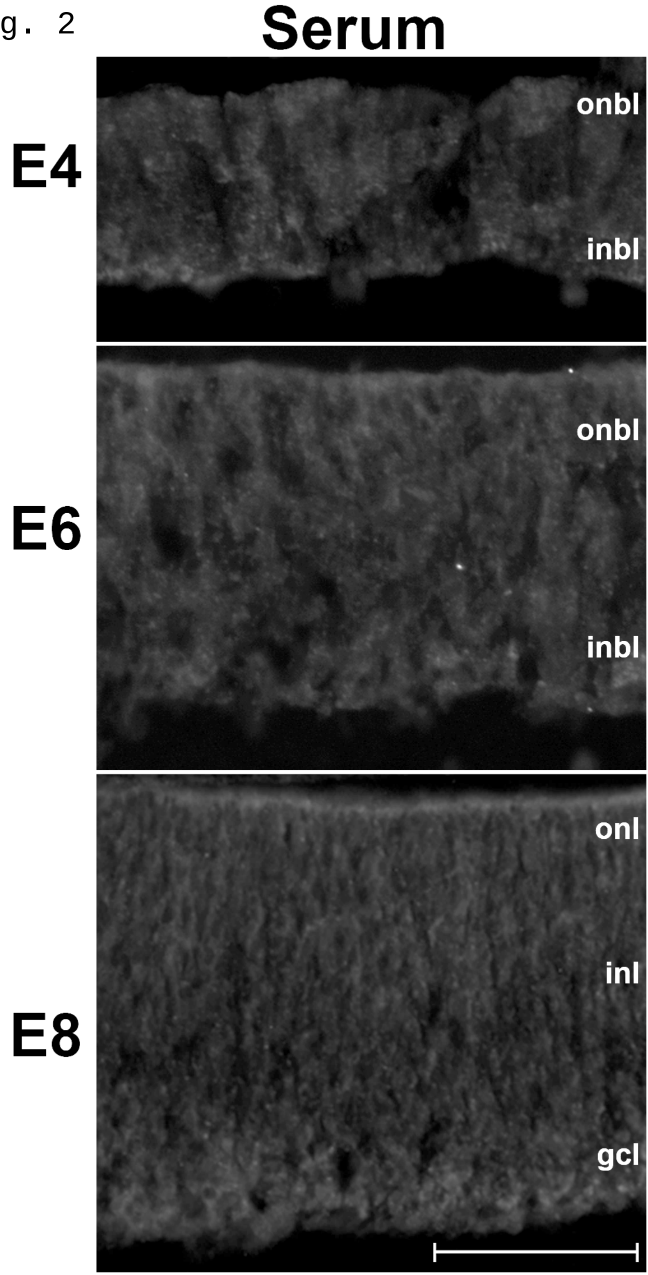

Supplement: Additional file 1 — Fig. S1. Diagram depicting the central region of the retina included for analysis. A. For retinas at E8 and older, the peripheral regions of the cryoprotected retinas were removed to ensure only the central region of the retina was included for analysis. The face of a clock will be used to describe the regions of the whole retina. The retina was oriented such that from an overhead view the dorsal region was oriented at 12 o'clock and the ventral region to the 6 o'clock position. Once situated in this orientation, a first cut is made from the 2 o'clock to the 10 o'clock positions. A second cut is made from the 4 o'clock to the 8 o'clock positions (red dotted lines in Fig. 3H). A third cut from the 1 o'clock to the 5 o'clock positions; and a final cut from the 7 o'clock to the 11 o'clock positions. The resulting square piece in the center is designated as central retina region. B. For retinas younger than E8, the whole eye was sectioned along with the head at the horizontal plane. The central region (CR) of the retina was defined as the area that opposite to the lens. Fig. S2. Negative control for antibody staining. Retina tissue from chicken embryos were harvested at E4, E6, and E8, sectioned, and stained with only serum and secondary antibody. ONBL, outer neuroblastic layer; INBL, inner neuroblastic layer; ONL, outer nuclear layer; INL, inner nuclear layer; GCL, ganglion cell layer. Scale bar = 40 μm. [file 1471-213X-10-8-S1.PDF]
